# Letter in response to “COVID‐19, Virchow's triad and thromboembolic risk in obese pregnant women”

**DOI:** 10.1002/clc.23601

**Published:** 2021-03-24

**Authors:** Giuseppe Calcaterra, Pier Paolo Bassareo, Jawahar L. Mehta

**Affiliations:** ^1^ Postgraduate Medical School University of Palermo Palermo Italy; ^2^ University College of Dublin, Mater Misericordiae University Hospital Dublin Republic of Ireland; ^3^ Division of Cardiology University of Arkansas for Medical Sciences and the VA Medical Center Little Rock Arkansas USA

To the Editor

We appreciate very much the letter by Carbillon and colleagues emphasizing the presence of Virchow's triad in pregnancy during the ongoing coronavirus disease 2019 (COVID‐19) pandemic. Initial data suggested that overall pregnancy is not a significant risk factor for developing severe COVID‐19, but new studies hint the risk of thromboembolism may be elevated for those who are obese or overweight. Indeed, Carbillon analyzed the three pivotal factors described by Virchow in the genesis of thromboembolism, because all components of this classic triad contributed together to an increased thromboembolic risk during the course of COVID‐19.^1^, ^2^, ^3^

Hypercoagulability increases in pregnancy by five‐fold. Obesity is known to alter the antiviral immune response as suppressors of cytokine signaling are upregulated and type I and III interferon responses are delayed and blunted, leading to ineffective viral clearance. Adipocyte secretion of leptin is pro‐inflammatory and high circulating levels of leptin have been associated with more severe respiratory disease. Moreover, the systemic effect of COVID‐19 virus induces an overwhelming “inflammatory state.” Further, the virus induces endothelial dysfunction by creating a state of “endothelialitis.” As the French authors say “the gravid uterus mechanically compresses veins as its volume increases, while the action of progesterone induces a loss of tone of the vein wall, and these factors combine their effect to slow blood flows when the patient is hospitalized and immobilized.”^1^, ^4^

Maternal obesity has emerged as a key risk factor increasing susceptibility of pregnant women to severe COVID‐19 disease, and most authors have recommended thromboprophylaxis in all obese women hospitalized with COVID‐19. The risk–benefit ratio is in favor of anticoagulant prophylaxis, as soon as the diagnosis of COVID‐19 has been made. It appears to be a sound practice in clinical practice.^5^

Obesity rates among pregnant women have increased drastically worldwide over the last three decades.^6^ The risk of moms‐to‐be passing the virus to the fetus is a “still evolving” area of research. Fortunately, a vertical transmission to the fetus is rare due the absence of ACE receptors on the placenta.^7^

## CONFLICT OF INTEREST

The authors declare no potential conflict of interest.
